# Predictiting valproate toxicity in patients with mitochondrial disorders

**DOI:** 10.1186/1878-5085-5-S1-A140

**Published:** 2014-02-11

**Authors:** Viktoria Remenyi, Anna Kekesi, Klara Pentelenyi, Aniko Gal, Vivien Hársfalvi, Maria Judit Molnar

**Affiliations:** 1 Institute of Genomic Medicine and Rare Disorders, Semmelweis University, Budapest Hungary

## Objective

The polymerase DNA gamma (POLG1) gene has major role in the biogenesis of mitochondrial DNA (mtDNA). POLG1 gene alterations may result in secondary multiple deletions in the mtDNA. Mutations in the POLG1 gene have been described with wide range of mitochondrial diseases, such as PEO, Alpers syndrome, and Parkinsonism. Until now seven POLG1 mutations (L304R, A467T, G588D, Q879H, T885S, E1143G and Q1236H) have pharmacogenetic significance to associate with valproic acid (VPA) induced liver toxicity. VPA is an anticonvulsant and mood-stabilizing drug using in the treatment of epilepsy, bipolar disorder.

## Patients and methods

We have investigated 61 patients with mitochondrial disease (male: 18, female: 43, mean age: 39±24 years) harbouring multiple mtDNA deletions in their muscle tissue. The mtDNA deletions were screened with long PCR. The total coding regions of the POLG1 gene (23 exons) were sequenced by Sanger method (ABI Prism 3500).

## Results

Three mutations (A467T, Q1143G, Q1236H) of the six VPA toxicity associated mutations have been found in 18 cases (29, 5%). The most frequently pathogenic POLG1 mutation in Caucasian population is the A467T (c.1399 G>A in exon 7) was found in one 4-year-old boy in heterozygous form. The sister of this child had VPA induced fatal hepatotoxicity. The E1143G (c.3428 A>G in exon 21) polymorfism was detected in 7 cases (male: 2, female: 5, mean age: 44±19, 68) in heterozygous form. We have found in 10 patients (male: 2, female: 8, mean age: 42±23, 75) the Q1236H (c.3708 G>T in exon 23) substitution in 2 cases homozygous, and in 8 cases heterozygous form.

## Conclusion

In mitochondrial disorders there is a high prevalence of VPA toxicity predicting genomic alterations. It has a major role in the disease management of these patients. In such cases before VPA treatment genotyping should be recommended to prevent the fatal hepatotoxicity.
